# Mechanisms of active metabolites from traditional Chinese medicine in osteoarthritis: a critical review

**DOI:** 10.3389/fphar.2026.1786112

**Published:** 2026-03-23

**Authors:** Hui Li, Yongli Zhao, Peng Qi, Bo Zhang, Jilong Ma, Xingwen Xie, Ning Li

**Affiliations:** 1 Affiliated Hospital of Gansu University of Traditional Chinese Medicine, Lanzhou, China; 2 Gansu University of Traditional Chinese Medicine, Lanzhou, China

**Keywords:** active metabolite, cartilage, inflammation, mechanism, osteoarthritis, signaling pathways, traditional Chinese medicine

## Abstract

Osteoarthritis (OA) is a prevalent degenerative joint disease with multifactorial pathogenesis and no cure. Traditional Chinese Medicine (TCM) is widely used in OA management, and accumulating research suggests that bioactive metabolites (formerly termed active components) from TCM botanical drugs can modulate key pathological processes in OA. Traditional Chinese medicine has an important role in OA management owing to its relatively low incidence of adverse effects, affordability, and multi-target therapeutic actions. In this review, we systematically searched PubMed and Web of Science (2010–2025) for studies on TCM-derived metabolites in OA using keywords such as “osteoarthritis,” “Traditional Chinese Medicine,” “active constituent,” and “mechanism.” Relevant *in vitro*, *in vivo*, and clinical studies were included based on predefined criteria. We critically evaluated the pharmacological rigor of these studies (models, controls, and translational relevance) and synthesized their findings. Numerous TCM metabolites including flavonoids, polyphenols, saponins, alkaloids, and polysaccharides exhibit chondroprotective effects in preclinical OA models by regulating inflammatory mediators, oxidative stress, cartilage matrix degradation, chondrocyte apoptosis senescence, macrophage polarization, ferroptosis, and gut microbiota composition. These compounds act through pathways such as NF-κB, PI3K/Akt/mTOR, and Wnt/β-catenin, reflecting multi-target mechanisms. Notably, compounds like quercetin and resveratrol are ubiquitous plant metabolites (not unique to TCM) and illustrate structure activity relationships. While promising, most evidence is confined to cell and animal studies with limited clinical validation. We discuss the strengths and limitations of current studies and identify priorities for future research, such as improving bioavailability and conducting high-quality clinical trials. In conclusion, TCM-derived metabolites show potential as multi-target agents for OA, but rigorous clinical studies and advanced delivery strategies are needed to translate these mechanistic insights into effective therapies.

## 1 Introduction

Osteoarthritis (OA) is among the most prevalent orthopedic disorders and is characterized by progressive degeneration of the entire joint, including articular cartilage, synovium, and subchondral bone (Sacitharan, 2019). Its pathogenesis is multifactorial, involving inflammation, oxidative stress, joint injury, aging, and other contributors. It is estimated that approximately 300 million people worldwide suffer from OA, which commonly leads to chronic joint pain, stiffness, and reduced quality of life (Murphy et al., 2018). OA is a major cause of disability in older adults and imposes a substantial economic burden, with annual healthcare costs in the United States alone reaching as high as USD 303 billion (Lambova and Müller-Ladner, 2018).

Current clinical management of OA is primarily symptomatic, focusing on pain relief and improved function (Li et al., 2017). Conservative treatments (e.g., oral nonsteroidal anti-inflammatory drugs and analgesics) may alleviate symptoms but are associated with significant adverse effects and do not prevent disease progression (Coppola et al., 2024). Apart from surgical joint replacement for end-stage OA, no approved therapy can halt or reverse cartilage degeneration. Therefore, there is an urgent need to identify safe, affordable, and effective disease-modifying treatments for OA (Zhao et al., 2024).

Traditional Chinese Medicine (TCM) is commonly used as an adjuvant approach in OA management in East Asia, supported by extensive clinical experience and a growing body of research. Traditional Chinese medicine exhibited distinct advantages in OA prevention and treatment through multi-pathway and multi-target actions. In China, TCM therapies including oral botanical drugs, topical herbal applications, and acupuncture have been widely used for OA (Liu CY. et al., 2024). A recent systematic review of 23 randomized controlled trials (2,175 patients with knee OA) reported that curcumin-based TCM interventions, either alone or combined with conventional therapy, significantly reduced pain and improved joint function with fewer adverse effects (Llamasares-Castillo et al., 2024). Another meta-analysis found that acupuncture also effectively alleviated OA pain and improved function (Kim et al., 2025). These clinical findings provide some support for TCM’s efficacy and safety in OA, although the precise active components and mechanisms underlying these benefits remain to be clarified.

Active constituents of TCM (typically secondary plant metabolites) are believed to form the principal material basis for TCM’s therapeutic effects. These compounds with defined chemical structures and known targets have become a major focus of modern pharmacological research (Wu et al., 2022). Increasing evidence indicates that TCM extracts and bioactive metabolites exert anti-OA effects via multiple mechanisms (Chen GY. et al., 2022). Reported actions include regulation of inflammatory mediator production, modulation of the gut microbiota, attenuation of oxidative stress, inhibition of cartilage matrix-degrading enzymes, regulation of immune cell (macrophage and lymphocyte) and chondrocyte metabolism, suppression of chondrocyte ferroptosis, and interference with OA-related signaling pathways (Chen GY. et al., 2022). For example, certain compounds such as baicalin and resveratrol display both anti-inflammatory and antioxidant activities, conferring direct chondroprotection while indirectly shaping the synovial immune milieu (Liang et al., 2024). Likewise, quercetin and icariin can act on chondrocytes and also modulate the behavior of other joint cells to exert integrated therapeutic effects. It should be noted that some of these metabolites (e.g., quercetin, resveratrol) are not unique to TCM but are ubiquitous in many plants and diets; their inclusion in this review is based on their relevance to TCM formulations and OA models.

Nevertheless, despite promising results, the precise active components and detailed mechanisms of TCM in OA are still not fully elucidated (Zhou X. et al., 2025). Many studies have been descriptive, and few have critically addressed pharmacological rigor or translational feasibility. Therefore, a critical review of current evidence is warranted. Based on the available literature, this review aims to systematically summarize recent progress in understanding how TCM-derived active metabolites prevent and treat OA, emphasizing mechanistic insights and the quality of evidence. We specifically focus on the molecular targets and pathways influenced by these compounds, discuss structure activity relationships where applicable, and critically evaluate the strengths and limitations of each study. Through this, we hope to provide a scientific basis and new ideas for further investigation of TCM metabolites as potential OA therapeutics.

## 2 Materials and methods

Literature Search: We performed a comprehensive literature search to identify studies on the mechanisms of TCM-derived metabolites in OA. The databases PubMed and Web of Science were searched for the period January 2010 to June 2025. The search strategy used various combinations of keywords, including “osteoarthritis,” “traditional Chinese medicine,” “herbal,” “active constituent,” “compound,” “metabolite,” and “mechanism.” We also searched by specific metabolite names and plant names associated with OA in TCM.

Inclusion and Exclusion Criteria: We included original research articles (in English or with English abstracts) reporting *in vitro* studies, animal experiments, or clinical trials that investigated the effects and mechanisms of single compounds or defined extracts derived from TCM botanical drugs on OA or OA-related molecular targets. Studies had to provide mechanistic insights (e.g., effects on inflammatory cytokines, enzymes, signaling pathways, etc.). We excluded review articles, studies on multi-herb formulations without focus on specific components, and studies lacking mechanistic endpoints.

Data Extraction and Synthesis: Relevant data from each eligible study were extracted, including the compound or extract investigated, its plant source, study design (cell type or animal model, intervention dosage and duration), key findings on mechanisms of action, and any noted study limitations. We paid special attention to whether studies included appropriate controls (e.g., positive controls like standard drugs, or negative controls), the concentrations or doses used relative to realistic pharmacological levels, and any translational considerations discussed. The findings were then organized by mechanistic themes (e.g., anti-inflammatory effects, regulation of oxidative stress, etc.) for synthesis. Throughout the Results and Discussion, we critically appraise the evidence by noting the rigor of study designs, consistency between different models, and potential biases or gaps.

## 3 Results and discussion

After screening, we identified numerous bioactive metabolites from TCM with reported anti-OA effects. The major categories of mechanisms, along with representative compounds, are discussed below. For each compound, we indicate the study model (*in vitro* and/or *in vivo*), main mechanistic findings, and consider the reliability and relevance of the evidence. Key information is also summarized in Table 1, including each metabolite’s source (validated plant species), chemical class, experimental model, and mode of action.

**TABLE 1:** Mechanisms of representative TCM-derived metabolites in osteoarthritis models.

Metabolite	Plant Source	Category	Model	Reported mechanism of action	References
Scoparone	Artemisia pubescens	Coumarins	Chondrocytes	Inhibition of iNOS and COX-2 in chondrocytes attenuates IL-1β-induced activation of PI3K/Akt/NF-κB pathway	Lu et al. (2018)
Ginsenoside Rg1	Ginseng, Panax notoginseng	Triterpenoid saponins	OA model rats	Inhibition of COX-2 and PGE2 secretion in chondrocytes	Cheng et al. (2017)
Tenuigenin	polygala tenuifolia	​	Human OA chondrocytes	Alleviate the release of NO, PGE2 and MMPs from chondrocytes	Wang et al. (2016)
Sclareol	Salvia Sclarea	Diterpenes	OA model rats	Altering bacterial abundance and regulating lipid metabolites	Jie et al. (2025)
Pterostilbene	Polygonum cuspidatum	Polyphenols	OA model mice	Changes in gut microbiota by reducing inflammation-related microbiota and increasing the number of healthy bacteria in OA group	Lee et al. (2024)
Gallic acid	rhubarb, dogwood	Polyphenols	OA model rats	Restore the abundance of anti-inflammatory flora in the intestinal tract of model rats, reduce the abundance of pro-inflammatory flora, and restore the balance of intestinal flora	Kang et al. (2024)
Quercetin	Ginkgo biloba leaves and sophora flower buds	Flavonoid	RAW264.7 cells	Polarization of macrophages to M2 macrophages promotes tissue repair	Philpott et al. (2025)
Fargesin	Wang Chunhua, Yulan	Lignans	OA model mice	Polarized M1-type Macrophage Cells Transformed into M2-type Cells *via* p38/ERK MAPK and p65/NF-κB Pathways	Lu et al. (2021)
Baicalin	Scutellaria baicalensis	Flavonoid	OA rats	Decreased the generation of reactive oxygen species in OA rats	Chen et al. (2018)
Dendrobine	dendrobe	Pyrrolidine derivatives	chondrocytes	Reduces intracellular reactive oxygen species production and inhibits IL-1 beta-induced activation of the NF-κB pathway	Chen et al. (2023)
Magnolin	magnolia	Lignan	OA rats	Restoration of TNF-α-induced upregulation of IL-1β, COX-2, ADAMTS-5 and MMP-1/3/13, and downregulation of chondrogenic ECM synthesis, thereby inhibiting cartilage matrix degradation, inhibiting MMP-13 expression, and promoting cartilage matrix construction	Xu et al. (2021)
Biochanin A	red clover	Oxymethylated isoflavones	rat chondrocytes	Reduce the increased expression of MMPs in chondrocytes, reduce cartilage degradation in OA rabbit models, and improve catabolic imbalance of cartilage matrix	Wu et al. (2014)
Alpha-mangostin	mangosteen	Xanthones	OA rats	Decrease INOS, COX-2, MMP-3, MMP-9 and the expression of MMP-13	Pan et al. (2017)
Quercetin	Ginkgo biloba leaves and sophora flower buds	Flavonoid	chondrocytes	Decreased pro-inflammatory markers (MMP9 and COX-2) and restored cartilage repair genes SOX9 and collagen type II	Lu et al. (2025)
Pterostilbene	Polygonum cuspidatum, resveratrol	Non-flavonoid polyphenols	Chondrocytes, OA mice	Alleviate chondrocyte injury, improve cell viability, reduce apoptosis rate	Zhou et al. (2025b)
Protocatechuic aldehyde	danshen	Natural polyphenol compound	chondrocytes	Improve SASP proteins such as MMP-3 and MMP-13 Expression level	Jie et al. (2024)
Biochanin A	Astragalus	Oxymethylated isoflavones	Chondrocytes, OA mice	Reduce iron deposition and severity of KOA, rescue cartilage cells killed by iron	He et al. (2023)
Paeoniflorin	Red peony root, white peony root	Monoterpene bitrin	chondrocytes	upregulation of SLC7A11 and GPX4 expression, inhibition of apoptosis of iron cells and reduction of iron deposition	Wu et al. (2025)
Salidroside	Rhodiola rosea	Flavonoid glycosides	chondrocytes	Attenuate chondrocyte apoptosis and oxide accumulation, protect the integrity of cartilage extracellular matrix, reduce cartilage (Nieuwenhuizen et al., 2013) cell apoptosis	Zhang et al. (2025)
Oroxin B	semen oroxyli	Flavonoid	chondrocytes	Inhibit the activation of PI3K/AKT/mTOR signaling pathway induced by IL-1β	Lu et al. (2022)
Icariin	herba epimedii	Flavonoid glycosides	SW1353 cells	Reduced phosphorylation of PI3K, Akt and mTOR, increased production of ULK1 and induced autophagy	Chen et al. (2022b)
Scutellarin	Scutellaria baicalensis	Flavonoid	SW1353 cells	Decreased protein expression levels of AKT, phosphorylated (p) AKT, mTOR and p-mTOR in the PI3K/AKT/mTOR signaling pathway in SW1353 cells	Ju et al. (2021)
Polygonatum sibiricum polysaccharides	Polygonatum odoratum	Polysaccharide compounds	OA model mice	Inhibits activation of TLR2/NF-κB signaling pathway in knee cartilage of OA mice and reduces serum inflammatory cytokine levels	Kuang et al. (2024)
Wedelolactone	herba ecliptae	Coumarins	OA model mice	Binding to NF-κB complex, thereby inhibiting nuclear localization of p65	Sun et al. (2024)
Resveratrol	resveratrol	Flavonoid	chondrocytes	Inhibition of IκB-α degradation and IL-1β-induced activation of NF-κB, inhibition of inflammation and matrix degradation in chondrocytes	Yi et al. (2020)
Achyranthes bidentata polysaccharide	achyranthes root	Polysaccharides	chondrocytes	Activation of Wnt/β-catenin signaling pathway promotes chondrocyte proliferation	Weng et al. (2014)
Icariin	herba epimedii	Flavonoid glycosides	SW1353 cells	Reduced phosphorylated p38, phosphorylated JNK and β-catenin levels	Zeng et al. (2014)
Artemisinin	sweet wormwood	Sesquiterpene lactones	chondrocytes	Inhibition of OA progression and cartilage degradation *via* Wnt/β-catenin signaling pathway	Zhong et al. (2018)

### 3.1 Regulation of immune and inflammatory responses

Evidence suggests that T lymphocytes promote extracellular matrix (ECM) degradation and remodeling by secreting multiple cytokines and growth factors, whereas B lymphocytes regulate chondrocyte ECM degradation through the release of autoantibodies and pro-inflammatory cytokines (Li et al., 2022). Following initial cartilage injury, cartilage-specific autoantigens are released, leading to infiltration of immune cells (including T and B cells) into joint tissues. Cytokines and chemokines are secreted by various cell types within the joint, and cartilage-degrading factors such as matrix metalloproteinases (MMPs) and prostaglandin E_2_ (PGE_2_) are activated and released, thereby exacerbating cartilage destruction (Haseeb and Haqqi, 2013). It has been reported that macrophages, T cells, and mast cells constitute the main infiltrating immune populations in synovitis associated with knee OA. Common pro-inflammatory cytokines, including tumor necrosis factor-α (TNF-α) and interleukin-1β (IL-1β), drive cartilage from a homeostatic state toward a catabolic phenotype and accelerate ECM breakdown (Kandahari et al., 2015). Inflammation is closely related not only to clinical symptoms but also to progressive cartilage loss, and pro-inflammatory mediators promote OA progression by disrupting normal chondrocyte function (Shen et al., 2017).

Interleukin-1β and TNF-α are widely regarded as two key catabolic inflammatory cytokines that induce cartilage degradation (Wu et al., 2020). Their overexpression in the joint activates downstream pathways such as nuclear factor-κB (NF-κB), thereby upregulating inflammatory mediators and matrix-degrading enzymes, including inducible nitric oxide synthase (iNOS), cyclooxygenase-2 (COX-2), MMP-3, MMP-13, and a disintegrin and metalloproteinase with thrombospondin motifs (ADAMTS), creating a vicious cycle of cartilage catabolism (Jeon et al., 2024). In addition, NF-κB signaling interacts with other pathways, further aggravating cartilage degeneration.

Several TCM-derived metabolites have demonstrated the ability to modulate these inflammatory cascades. For example, scoparone is a coumarin compound (6,7-dimethoxycoumarin) derived from *Artemisia capillaris* Thunb. (also known as *Herba Artemisiae Scopariae*). In an *in vitro* study using IL-1β–stimulated human chondrocytes, (Lu et al., 2018) reported that scoparone (at 50–200 µM) dose-dependently suppressed IL-1β induced expression of inducible nitric oxide synthase (iNOS) and cyclooxygenase-2 (COX-2), resulting in reduced NO and PGE_2_ production. Scoparone also attenuated the excessive activation of the PI3K/Akt/NF-κB signaling pathway in these chondrocytes. Through these actions, scoparone effectively blocked the IL-1β mediated inflammatory response and protected cartilage matrix *in vitro*. Critically, this evidence comes from a cellular model:while it demonstrates a mechanism (inhibition of NF-κB and downstream mediators), the study did not include an animal model or *in vivo* data. Thus, the therapeutic potential of scoparone in OA remains hypothetical until supported by animal or clinical studies.

Another example is ginsenoside Rg1, a triterpenoid saponin isolated from *Panax ginseng*. and *Panax notoginseng*. Cheng et al. (Cheng et al., 2017) investigated Rg1 in an anterior cruciate ligament transection (ACLT) rat model of OA. Rats received Rg1 (5, 10, or 20 mg/kg) by oral gavage for 8 weeks. The treatment was found to ameliorate cartilage degeneration in a dose-dependent manner. Specifically, Rg1-treated rats showed reduced levels of COX-2 and PGE_2_ in joint tissues and decreased degradation of type II collagen and aggrecan (key cartilage ECM components) compared to untreated OA controls. These findings suggest that Rg1 exerts significant anti-inflammatory and chondroprotective effects *in vivo*. It is noteworthy that this study included both *in vitro* experiments on human OA chondrocytes (to confirm mechanistic targets) and the *in vivo* rat model for disease relevance. The use of a surgical OA model (ACLT) strengthens the relevance to post-traumatic OA, and the dose-dependent response adds credibility. However, the study did not compare Rg1 to existing OA drugs (e.g., NSAIDs) as a positive control, leaving open the question of how Rg1’s efficacy and safety profile might compare to standard treatments. Additionally, ginsenosides like Rg1 are known to have poor oral bioavailability; whether sufficient levels reach the joint to be therapeutic in humans is uncertain. These issues highlight the need for further pharmacokinetic and possibly clinical studies on Rg1.

Tenuigenin, a major bioactive metabolite from *Polygala tenuifolia Willd*, has also shown anti-inflammatory activity in OA contexts. Wang et al. (2016) examined tenuigenin in IL-1β–stimulated human OA chondrocytes *in vitro*. Tenuigenin at 2, 4, and 8 µM significantly alleviated the inflammatory response: it reduced NO and PGE_2_ release and lowered MMP-3 and MMP-13 production by downregulating the PI3K/Akt/NF-κB pathway. These concentrations of tenuigenin are relatively low (micromolar range), suggesting potency. However, no *in vivo* experiments were reported in this study. Thus, while tenuigenin can suppress catabolic inflammation in isolated chondrocytes, its efficacy in an organism (and its bioavailability to joint tissues) remains to be determined. This is a common limitation for many such studies: promising *in vitro* results must be interpreted with caution without animal model validation.

### 3.2 Modulation of gut microbiota dysbiosis

The gut microbiota can influence bone homeostasis by regulating osteoclasts and osteoblasts through immune, endocrine, and metabolic systems (Li et al., 2021). Dysbiosis may disrupt the intestinal mucosal barrier, increase gut permeability, and allow microbial products, intestinal antigens, and pro-inflammatory factors to enter systemic circulation, thereby activating innate and adaptive immunity and exacerbating knee osteoarthritis (Sun et al., 2023). Probiotics can improve host microecological balance. Short-chain fatty acids, key microbial metabolites produced by bacterial fermentation of dietary fiber, play important roles in intestinal metabolism and barrier integrity (Cho et al., 2022). Studies have shown that administration of live lactobacilli and butyrate in OA model rats improved the gut microenvironment and restored abnormal autophagy, alleviating pain and protecting articular cartilage (Lian et al., 2022). The gut microbiota plays a crucial role in regulating autophagy, oxidative stress, and inflammation in knee OA. On the one hand, microbial communities can modulate mitochondrial regulators such as AMPK activity and PGC-1α signaling; on the other hand, butyrate can reduce inflammation, diminish reactive oxygen species production, and reverse ECM degradation in inflamed chondrocytes.

Some TCM metabolites appear to beneficially alter the gut microbiota in OA. Sclareol (SCL) is a diterpenoid alcohol from *Salvia sclarea L*. (clary sage) with known anti-inflammatory and antimicrobial properties. Jie et al. (2025) treated rats in a knee OA model (induced by medial meniscus destabilization, as described in the study) with oral sclareol and investigated its impact on the gut microbiome and joint inflammation. Sclareol administration led to shifts in gut bacterial composition: for example, it increased beneficial bacteria in the Prevotellaceae family and Corynebacterium genus while modulating levels of lipid metabolites relevant to inflammation. To establish causality, the researchers performed fecal microbiota transplantation (FMT): germ-free or antibiotic-treated recipient rats received fecal microbes from sclareol-treated OA rats. Interestingly, the FMT from sclareol-treated donors was sufficient to reduce synovial inflammation and macrophage necroptosis in recipient OA rats. This suggests that sclareol’s benefits are at least partly mediated by the microbiota and its metabolites. From a critical standpoint, this combined *in vivo* and *ex vivo* approach is robust, reinforcing the mechanistic link between gut microbiota changes and joint protection. One limitation, however, is that the exact bacterial strains or metabolites responsible were not identified a common challenge in microbiome studies. Also, the OA model was in rats, and similar gut-joint interactions need verification in human OA. Nonetheless, sclareol’s relatively high oral bioavailability and effect on the “gut–bone axis” highlight it as an intriguing candidate for further research.

Pterostilbene (PT) provides another example; it is a natural polyphenol (structurally a dimethylated analog of resveratrol) found in blueberries and also in some TCM herbs (*e.g.*, *Darach*, possibly referring to *Pterocarpus* genus). Compared to resveratrol, pterostilbene’s two methoxy substitutions increase its lipophilicity and metabolic stability. Lee et al. (2024) evaluated pterostilbene in a mouse OA model (induced by obesity-related inflammation) and in IL-1β–stimulated chondrocyte cultures. Pterostilbene treatment suppressed activation of the NLRP3 inflammasome and inhibited NF-κB signaling, thereby preventing excessive production of inflammatory cytokines and catabolic enzymes in joint tissues. Notably, PT also modulated the gut microbiota: it reduced the abundance of inflammation-associated microbial taxa (e.g., certain *Proteobacteria*) and increased beneficial commensals. These microbiota changes correlated with improved systemic inflammation markers in the PT-treated mice. The study’s strength lies in its integration of mechanistic analyses (inflammasome, NF-κB) with microbiome profiling, suggesting a multi-faceted mode of action for pterostilbene. Additionally, the authors assessed safety, finding no significant toxicity with long-term PT administration in mice. However, one should consider that pterostilbene’s effective dose in mice may be high relative to achievable human intake, and that rodent microbiomes differ from humans. Follow-up studies could examine whether lower doses or pterostilbene-rich diets have similar effects, and potentially a clinical trial for biomarkers of inflammation.

Gallic acid, a simple phenolic metabolite present in various TCM botanicals such as *Rheum palmatum* L. (rhubarb) and *Cornus officinalis*, has known antioxidant and anti-inflammatory properties. Kang et al. (2024) administered gallic acid to rats with surgically induced OA and analyzed gut microbial changes. They observed that gallic acid markedly increased the relative abundance of anti-inflammatory gut bacteria (such as *Lactobacillus* spp.) and decreased that of pro-inflammatory bacteria. Moreover, gallic acid altered fecal metabolic profiles, particularly fatty acid and amino acid metabolites that can influence inflammation. Correspondingly, the treated rats showed reduced synovial inflammation and fibrosis. This study is valuable in linking a well-known dietary polyphenol to gut-mediated anti-OA effects. However, it remains somewhat descriptive–while correlations between microbiota shifts and joint improvements are shown, direct causal evidence (such as FMT experiments) was not provided. Additionally, gallic acid is not unique to TCM (it’s found in many fruits and teas), so its effects are not surprising given literature in other inflammatory conditions. A critical takeaway is that systemic metabolic effects of oral polyphenols (through the gut microbiome or direct absorption) may contribute significantly to their anti-OA activity, beyond direct action in the joint.

### 3.3 Regulation of macrophages

Macrophages are key immune cells within the joint and play critical roles in shaping the local immune microenvironment and promoting tissue repair. After joint injury, synovial macrophages tend to polarize toward the pro-inflammatory M1 phenotype, driving increases in IL-1β, TNF-α, NO, and PGE2, exacerbating OA synovitis, enhancing MMP production in synovial fibroblasts, and accelerating cartilage matrix degradation (Hsueh et al., 2021). In contrast, increased expression of M2 macrophage markers such as CD163, arginase-1, and the mannose receptor promotes the release of anti-inflammatory cytokines (e.g., IL-4, IL-10, transforming growth factor-β, and insulin-like growth factors), enhances type II collagen synthesis, drives macrophage polarization toward the M2 phenotype, facilitates the clearance of matrix-degrading enzymes, and promotes tissue remodeling (Orlowsky and Kraus, 2015).

Several TCM metabolites have been reported to influence macrophage polarization to favor the M2 state. Quercetin is a ubiquitous flavonoid found in many plants (in TCM context, notable sources include *Ginkgo biloba* L. leaves and *Sophora japonica* L. flower buds, known as Sophorae Flos). Quercetin is known for its antioxidant and immunomodulatory activities. In an *in vitro* study, (Philpott et al., 2025) treated RAW264.7 macrophages with quercetin (8 µM) and observed activation of the Akt/STAT6 pathway, which promoted nuclear translocation of p-STAT6 and p-Akt. As a result, quercetin induced polarization of these macrophages toward an M2-like phenotype: expression of M2 markers (Arg1, CD206/MR, Ym1) and secretion of chondrogenic growth factors (IGF-1, TGF-βs) increased, while M1 markers (CD68) and pro-inflammatory cytokines (TNF-α, IL-1β, iNOS) were not upregulated. This suggests that quercetin can reprogram macrophages to a reparative state, which could indirectly benefit cartilage. Consistent with the *in vitro* findings, an *in vivo* study by Philpott et al. (2025) in a rat OA model found that quercetin administration reduced synovial inflammation and apoptosis and increased the prevalence of M2 macrophages in the joint. Treated rats had improved cartilage histology compared to controls. From a critical perspective, quercetin’s dual activities on both chondrocytes (directly reducing oxidative and inflammatory damage) and synovial macrophages (modulating the microenvironment) make it a particularly interesting candidate. However, quercetin’s poor bioavailability is a known challenge–high oral doses or specialized delivery systems might be required to achieve effective joint levels. The *in vivo* study by Philpott et al. used quercetin aglycone intragastrically at a dose that, while effective in rats, might not translate easily to humans without formulation improvements.

Fargesin is a lignan metabolite extracted from the flower buds of *Magnolia* species (including *Magnolia biondii*, *M. denudata*, *M. sprengeri* in TCM, collectively called Xin Yi). Lu et al. (2021) found that fargesin (10 mg/kg) partially suppressed excessive activation of the p38/ERK MAPK and p65/NF-κB pathways in cartilage. It reduced the numbers of F4/80-positive cells (macrophage marker) and iNOS-positive cells (M1 marker) in synovial tissue, while increasing CD206-positive cells (M2 marker), indicating a higher proportion of M2-polarized synovial macrophages. Concurrently, fargesin decreased the expression of matrix-degrading enzymes such as MMP-13 and type X collagenase, thereby alleviating cartilage destruction. Further cellular experiments showed that fargesin (0–40 μmol/L) inhibited p38/ERK MAPK and p65/NF-κB signaling, reprogrammed M1-polarized macrophages toward an M2 phenotype, upregulated M2 markers (e.g., CD206) and anti-inflammatory IL-10, and reduced LPS-induced iNOS, IL-1β, and IL-6. These results indicate that fargesin may relieve local joint inflammation by remodeling synovial macrophage functional phenotypes, thereby exerting anti-OA effects. A limitation to note is that ip injection bypasses first-pass metabolism; the effective oral dose might differ. Nonetheless, fargesin’s ability to “reprogram” macrophages and simultaneously reduce chondrocyte catabolic signaling presents a strong case for further development. Future studies might explore nanoformulations to enhance its delivery to joint tissues or test it in combination with other agents.

### 3.4 Attenuation of oxidative stress

Reactive oxygen species (ROS) play a pivotal role in the initiation and progression of OA. Excess ROS within the joint accelerates oxidative stress, causing severe damage to chondrocytes and the ECM and thereby promoting OA progression (Ansari et al., 2020). Hydrogen peroxide (H_2_O_2_) is a common ROS form, and its accumulation contributes to OA development. It induces iNOS expression in human chondrocytes, and iNOS is a key enzyme for nitric oxide (NO) synthesis (Fitzpatrick and Athwal, 2020). Once induced, NO can be produced at a constant rate over extended periods, inhibiting type II collagen synthesis and promoting its degradation (Xia et al., 2014). Nitric oxide also suppresses ECM secretion by chondrocytes, disrupts nutrient exchange within cartilage, and maintains chondrocytes in an unfavorable microenvironment, ultimately leading to ECM degradation and gradual cartilage degeneration (Abramoff and Caldera, 2020). In addition, malondialdehyde and superoxide dismutase (SOD) are two important indicators of oxidative stress and participate in intracellular signaling; imbalance in their levels exacerbates oxidative stress (Marchev et al., 2017).

TCM is rich in antioxidant metabolites, and some have been tested in OA contexts. Baicalin is a flavonoid glycoside (baicalein 7-O-glucuronide) extracted from the roots of *Scutellaria baicalensis* Georgi. It exhibits both antioxidant and anti-inflammatory activities. Chen et al. (2018) evaluated baicalin in an experimental rat OA model induced by knee immobilization and also examined its effects on skeletal muscle around the joint. Rats treated with baicalin (100 mg/kg ip) showed reduced joint pain behavior and improved muscle function compared to OA controls. Mechanistically, baicalin upregulated the Nrf2 pathway in muscles and cartilage, leading to increased expression of antioxidant enzymes. It also decreased the production of IL-6 and other inflammatory mediators and attenuated excessive ROS accumulation in joint tissues. Histologically, baicalin-treated rats had less cartilage erosion and more normal muscle fiber morphology than untreated OA rats. From a critical standpoint, this study is interesting because it looks beyond cartilage, recognizing that OA often involves periarticular muscle weakness (which can be exacerbated by oxidative stress). By demonstrating that baicalin can simultaneously protect cartilage and muscle via an antioxidant mechanism, it broadens the potential therapeutic impact. However, one limitation is the OA model (joint immobilization is a non-traditional model that induces disuse atrophy and cartilage changes, which may differ from idiopathic OA). Also, the systemic delivery of baicalin by injection might not reflect typical oral herbal use; baicalin’s oral bioavailability is limited (it is largely converted to its aglycone baicalein and conjugates). Future work could examine if oral *Scutellaria* extracts (which contain baicalin) have similar effects, and how baicalin compares to other antioxidants or exercise in preserving muscle function.

Dendrobine is an alkaloid isolated from *Dendrobium nobile* Lindl. (Shi Hu, an orchid used in TCM). Chen et al. (2023) investigated dendrobine’s effects on chondrocyte aging and OA. *in vitro*, dendrobine (at ∼10 µM) protected IL-1β stimulated chondrocytes by reducing intracellular ROS production and suppressing NF-κB activation. The authors also used a ROS scavenger (N-acetylcysteine, NAC) as a comparison, and found that NAC and dendrobine both blocked IL-1β–induced NF-κB nuclear translocation, suggesting dendrobine works largely by mitigating ROS signaling. Additionally, in a D-galactose-induced aging rat model (which causes oxidative stress and a premature aging-like condition in multiple tissues including joints), dendrobine treatment reduced markers of cellular senescence and cartilage degeneration (data from that model were mentioned in the text). This dual *in vitro*/*in vivo* approach strengthens confidence in dendrobine’s antioxidant chondroprotective effect. A notable insight is that by lowering ROS, dendrobine indirectly kept NF-κB (a redox-sensitive transcription factor) inactive, thus reducing downstream inflammation. Limitations include that the OA relevance of the D-galactose model is indirect; it models aging rather than cartilage trauma or obesity-related OA. Nonetheless, since age is a major OA risk factor, interventions that target aging mechanisms (like oxidative stress) are valuable. Dendrobine is a relatively unique scaffold (a sesquiterpene alkaloid) not widely studied outside of certain contexts, so more work is needed to define its safety and pharmacokinetics.

### 3.5 Inhibition of matrix-degrading enzymes

The cartilage extracellular matrix is essential for regulating chondrocyte metabolism and function. Articular cartilage is avascular, alymphatic, and aneural, and chondrocytes are the only cellular component. The remaining structure consists of ECM, which is composed of water (>70%) and organic components such as type II collagen, proteoglycans, and other collagens (Wilusz et al., 2014). Under physiological conditions, cartilage constituents are organized within a complex, porous, and permeable ECM that provides unique fluid pressurization capacity, enabling long-term load-bearing. This remarkable function depends on the distinctive structural organization and mechanical properties of the ECM. During KOA pathology, a reduction in type II collagen disrupts the integrity of the ECM network formed by collagen and proteoglycans, progressively altering the biological and mechanical microenvironment of the joint (Maldonado and Nam, 2013). Excessive mechanical loading disrupts the balance between anabolism and catabolism, leading to depletion of ECM components. Given the limited regenerative capacity of cartilage, this results in irreversible damage and becomes a key driver of OA (Rahmati et al., 2017). Therefore, altered matrix synthesis, upregulated matrix-degrading proteins, and changes in the mechanical microenvironment accelerate ECM degradation, alter the mechanical properties of chondrocytes, aggravate cartilage destruction, and ultimately accelerate OA progression. The ECM is mainly composed of type II collagen and a large proteoglycan network containing glycosaminoglycans, hyaluronic acid, and chondroitin sulfate (He et al., 2022).

Matrix metalloproteinases are a family of zinc-dependent endopeptidases with broad substrate specificity. They can cleave fibrillar collagens and many ECM components, as well as target cell-surface molecules and extracellular proteins unrelated to ECM, thereby influencing cellular regulatory behaviors (Cui et al., 2017). Matrix metalloproteinases are generally secreted as inactive pro-MMPs and become activated through proteolytic cleavage. Extracellular matrix degradation is essential for processes such as embryonic development, angiogenesis, cell repair, and tissue remodeling. However, dysregulated MMP expression leads to abnormal ECM degradation and contributes to KOA pathology (Li and Wu, 2021). Overexpression of MMPs and ADAMTS, key enzymes driving cartilage matrix degradation, promotes breakdown of proteoglycans and collagen and accelerates OA progression. Tissue inhibitors of metalloproteinases (TIMPs) can inhibit proteoglycan activity and reduce proteoglycan degradation. In recent years, MMP-1, MMP-3, and other MMP family members have been most closely associated with KOA (Zeng et al., 2017).

TCM-derived metabolites that suppress MMP/ADAMTS expression or activity can thereby slow cartilage erosion. Magnolin (MGL) is a lignan isolated from *Magnolia* flower buds (the same source as fargesin). Xu et al. (2021) studied magnolin in an ACLT-induced OA rat model. They delivered magnolin via intra-articular injection (a route ensuring the compound reaches cartilage) at a certain concentration weekly for several weeks. Magnolin-treated knees showed significantly reduced levels of IL-1β, COX-2, ADAMTS-5, and MMP-1/3/13 in joint tissues compared to vehicle-treated OA knees. This was accompanied by higher expression of anabolic markers like collagen II and SOX9 (a chondrogenic transcription factor), indicating a restoration of cartilage matrix synthesis. Histologically, magnolin dramatically attenuated cartilage destruction; OARSI scores (a measure of cartilage degeneration) were much improved. The mechanism in chondrocytes was linked to NF-κB pathway inhibition: magnolin prevented the nuclear translocation of NF-κB p65, thereby blocking the transcription of MMP genes. This study presents a compelling case for magnolin as a potent chondroprotective agent. The intra-articular delivery is a strength in demonstrating local efficacy, though it might not reflect the common usage (most herbal treatments are oral). It would be interesting to know magnolin’s effectiveness if given orally or systemically, as intra-articular injection is an invasive route. Also, no toxicity was noted, but systemic safety would need evaluation if oral dosing is considered. Magnolin’s structural similarity to other magnolia lignans suggests it might share anti-inflammatory properties (magnolia extracts are known to have NF-κB inhibiting compounds). The specific benefit here is the focus on cartilage matrix preservation.

Biochanin A (BCA) is an O-methylated isoflavone abundant in red clover (*Trifolium pratense* L.) and present in small quantities in some Astragalus species. It has estrogenic and anti-inflammatory effects. Wu et al. (2014) reported that biochanin A at 10–50 µM dose-dependently inhibited IL-1β induced MMP-3, -9, and -13 expression in rat chondrocytes *in vitro*. Furthermore, in a rabbit ACLT OA model (rabbits were chosen for their larger joint size relative to rodents), intra-articular injection of biochanin A significantly reduced cartilage erosion and restored a more normal balance of matrix metabolism. Molecular analysis indicated that BCA suppressed NF-κB activation (again by preventing p65 translocation) in cartilage, which explains the reduced MMP production. The use of a rabbit model is less common but noteworthy, as rabbits develop OA changes that can be more severe and human-like (with pronounced cartilage ulceration). That BCA was effective in this model bodes well for its potency. However, like magnolin, BCA was delivered locally to the joint in this study. Orally, biochanin A is metabolized into other compounds (like genistein or phase II conjugates), so whether oral intake yields sufficient joint exposure is unclear. On the other hand, red clover extracts (rich in BCA and related isoflavones) are already used as dietary supplements (e.g., in menopausal symptoms) with a known safety profile, which could accelerate repurposing if efficacy is shown. Another consideration: isoflavones can have mild estrogenic activity, which might be beneficial for cartilage (estrogen has chondroprotective effects) but also could pose risks (e.g., for certain cancers). In OA, post-menopausal estrogen loss is linked with increased incidence, so an isoflavone like BCA might partly act through estrogen receptor pathways–an aspect not explored in the Wu et al. study but worth investigating.

Alpha-mangostin (α-MG) is a xanthone from the pericarp of mangosteen (*Garcinia mangostana* L.). Pan et al. (2017) assessed α-mangostin in both cellular and animal OA models. In IL-1β stimulated rat chondrocytes, α-MG (10 µM) reversed the induction of iNOS, COX-2, and MMP-3/-9/-13, preserving type II collagen and aggrecan levels. It markedly inhibited the phosphorylation and nuclear translocation of NF-κB p65 in these cells. Meanwhile, in a DMM (destabilization of the medial meniscus) mouse model, oral or ip α-MG treatment (the study used a certain mg/kg dose) led to significantly less cartilage degeneration and lower OARSI scores relative to untreated OA mice. These findings align with the pattern we see: α-MG likely exerts chondroprotection by suppressing NF-κB–mediated inflammation and matrix catabolism. The value of α-MG is that it’s a well-characterized molecule with known antioxidant/anti-inflammatory effects in other diseases (like colitis, cancer models). Its translation might be eased by existing data, but a potential hurdle is its lipophilicity, which can limit oral absorption. The study by Pan et al. does not detail pharmacokinetics, but observing efficacy *in vivo* suggests some bioavailability. Critically, α-MG’s effects in the DMM model (which mimics a common clinical scenario of meniscal injury leading to OA) underscore its relevance. In this model, α-MG not only improved histology but also presumably had an effect on pain (although not explicitly stated, often weight-bearing or pain behavior is assessed). If α-MG reduces pain by reducing synovitis and preserving cartilage, it could be a holistic treatment approach.

### 3.6 Regulation of chondrocyte metabolism

Cellular senescence is a cell fate characterized by permanent cell-cycle arrest with sustained metabolic activity and secretion of inflammatory factors into the tissue microenvironment, a phenomenon referred to as the senescence-associated secretory phenotype (SASP). Chondrocyte apoptosis is a hallmark of OA. Numerous genes and signaling pathways regulate chondrocyte proliferation and apoptosis; aberrant gene and pathway activation suppresses proliferation and promotes apoptosis. Autophagy helps maintain normal chondrocyte physiology, and its reduction aggravates chondrocyte injury (Li et al., 2020). Bone marrow mesenchymal stem cells possess strong differentiation capacity and can be induced to become chondrocytes under conditions favorable for cartilage reconstruction. Compared with normal chondrocytes, OA chondrocytes exhibit multiple senescence-associated features, including elevated ROS levels, increased β-galactosidase activity, upregulated cyclin-dependent kinase inhibitors p21, and secretion of inflammatory factors such as IL-6, TNF, and CXCL8 (Xu et al., 2017). In KOA, senescent and degenerative chondrocytes show reduced stress responsiveness and diminished anabolic capacity, leading to imbalance between ECM synthesis and degradation and subsequent cartilage degeneration (Huang et al., 2025). Moreover, senescent chondrocytes are not readily cleared by the immune system yet remain metabolically active, continuously secreting SASP into the joint cavity, thereby maintaining chronic pathological inflammation and promoting synovitis (Li et al., 2025). Senescence-associated secretory phenotype factors and extracellular vesicles released by senescent cells disrupt surrounding tissues and homeostasis via chronic sterile inflammation, induce OA-related alterations, and can promote senescence in neighboring normal cells (Nelson et al., 2012).

Traditional Chinese medicine metabolites have been explored for these purposes. Quercetin, revisited here in a different context, has limited oral bioavailability as an aglycone, but novel delivery methods are being investigated to target it to chondrocytes. One innovative approach by Lu et al. (2025) involved mesenchymal stem cell–derived exosomes (MSC-Exos) as carriers. Bone marrow MSCs were pretreated with quercetin (1 µM) so that they would load quercetin into exosomes naturally during exosome biogenesis. The isolated “Que-Exos” were then applied to IL-1β–treated chondrocytes. Compared to control exosomes, Que-Exos delivered quercetin into chondrocytes more effectively, resulting in a significant downregulation of pro-inflammatory markers (MMP-9, COX-2) and an upregulation of cartilage repair genes (SOX9, collagen II). In an ACLT mouse OA model, intra-articular injection of these quercetin-loaded exosomes led to improved Safranin O staining (indicating better glycosaminoglycan content) and lower OARSI scores than controls. This study is quite forward-looking, as it addresses two major issues: quercetin delivery and the concept of using MSC exosomes which themselves may aid cartilage regeneration. The results imply that local delivery of quercetin can indeed protect cartilage by enhancing anabolic responses and mitigating inflammation. The use of exosomes is complex and expensive, but it might not be necessary if simpler delivery (like nanoparticles or better formulations) could achieve similar local drug concentrations. An important insight is that combining a natural compound with modern delivery systems can potentially overcome bioavailability hurdles, a strategy that could be applied to many TCM metabolites.

Resveratrol, a stilbene polyphenol, has been reported to influence chondrocyte survival pathways. Zhou L. et al. (2025b) used an OA mouse model (likely induced by surgical instability) and cultured human OA chondrocytes to test resveratrol’s effects. Resveratrol (administered intra-articularly in mice at various doses) alleviated cartilage damage and improved cartilage morphology *in vivo*. At the cellular level, resveratrol reduced IL-1β induced inflammatory injury, improved chondrocyte viability (meaning it reduced cell death), decreased apoptosis markers, and even promoted autophagosome formation. Mechanistically, it appeared to modulate the Ras/Raf/MEK/ERK pathway, which is implicated in cell survival and apoptosis. The Ras–ERK pathway can promote apoptosis in chondrocytes if overactivated, so resveratrol’s modulation of this pathway may tilt the balance towards survival. Yi et al. (2020) also found that resveratrol (50 µM) inhibited IL-1β triggered NF-κB activation in human OA chondrocytes, thereby suppressing inflammation and matrix degradation. Taken together, resveratrol seems to have pleiotropic beneficial effects: anti-apoptotic, pro-autophagic, and anti-inflammatory. However, resveratrol suffers from low oral bioavailability (20% in humans) and rapid metabolism. The studies mentioned used either intra-articular injection or high concentrations *in vitro*. Clinically, oral doses of resveratrol (up to 1 g/day) have been given in trials for other indications, but achieving joint concentrations comparable to those *in vitro* might be difficult. Chemical modification (like in pterostilbene) or formulation (liposomes, etc.) might enhance its therapeutic potential for OA. Also, resveratrol is not TCM-specific; it’s more of a general natural compound, but *Polygonum cuspidatum* (a TCM herb) is a rich source of resveratrol, which justifies its inclusion in TCM discussions.

Another interesting compound is protocatechuic aldehyde (PA), an active phenolic component of *Salvia miltiorrhiza* Bunge (Danshen). Rather than focusing on classic inflammation, a study by Jie et al. (2024) examined PA’s effect on mitophagy (mitochondrial autophagy) and chondrocyte senescence. They found that PA (treatment of IL-1β exposed chondrocytes) upregulated PINK1/Parkin-mediated mitophagy, which is a pathway for clearing damaged mitochondria. By enhancing the removal of dysfunctional mitochondria, PA reduced the levels of SASP factors (like MMP-3, MMP-13, IL-6) in chondrocytes, effectively delaying cellular senescence and reducing the pro-inflammatory secretory profile. This suggests PA might help maintain a healthier population of chondrocytes by preventing the accumulation of senescent cells. In the same study, PA also suppressed NF-κB activation (consistent with many other compounds). While primarily an *in vitro* study, it highlights a less common angle: targeting cellular quality control processes (autophagy/mitophagy) to combat OA. Considering that mitophagy declines with age and that mitochondrial dysfunction is implicated in OA chondrocytes, compounds like PA offer a promising route to rejuvenate cell function. Clinically, Danshen extracts are already used in cardiovascular contexts, and PA is one of their major components, this could facilitate translational research if joint-targeted formulations are devised. However, detailed pharmacological data on PA specifically in joints are needed.

### 3.7 Inhibition of ferroptosis

Ferroptosis is a newly characterized form of regulated, non-apoptotic cell death, primarily marked by abnormal accumulation of lipid peroxides driven by iron-dependent reactions (Ru et al., 2024). At the molecular level, ferroptosis largely depends on impaired glutathione peroxidase 4 (GPX4) function (Zhang et al., 2024). The cystine/glutamate antiporter system Xc, composed of SLC7A11 and solute carrier family, is also a key regulator of ferroptosis (Yu et al., 2025). Evidence suggests that systemic iron overload and iron deposition in synovium and cartilage play crucial roles in KOA pathophysiology (Nieuwenhuizen et al., 2013). Iron is an essential trace element required for maintaining redox homeostasis and energy metabolism, and cartilage possesses dynamic mechanisms for iron homeostasis (Liu Y. et al., 2024). However, dysregulated iron metabolism leading to excessive labile ferrous iron generates harmful free radicals via chemical reactions, triggers oxidative stress, causes structural damage and inflammation, and ultimately impairs joint function (Wang et al., 2022). During OA progression, chondrocytes are exposed to sustained inflammatory stimulation and oxidative stress, resulting in gradual functional loss and even regulated cell death (Guan et al., 2024). Current studies show that OA cartilage displays markedly increased ROS levels along with downregulation of GPX4 and SLC7A11, suggesting activation of ferroptosis (Ruan et al., 2024). Glutathione peroxidase 4 is a key antioxidant enzyme that clears lipid peroxides; its inactivation or reduced expression leads to persistent accumulation of phospholipid peroxides on cell membranes, culminating in cell death (Xie et al., 2023). Meanwhile, reduced SLC7A11 expression weakens extracellular cystine uptake and glutathione synthesis, thereby diminishing antioxidant capacity and further driving irreversible ferroptosis (Chen et al., 2021).

Bioactive compounds from TCM that modulate ferroptosis have been identified. Biochanin A (BCA), introduced earlier for MMP inhibition, also has anti-ferroptotic effects. He et al. (2023) used an iron-overload OA model in mice (injecting iron into the joint to exacerbate OA). Mice receiving BCA had less cartilage degeneration on micro-CT and histology, and importantly, had reduced iron deposition in the cartilage and synovium compared to iron-only controls. in *vitro*, BCA protected primary chondrocytes from ferric ammonium citrate (FAC)-induced ferroptosis: it lowered intracellular free iron levels, reduced ROS and lipid peroxidation, and preserved mitochondrial membrane potential (as shown by JC-1 staining). The study also noted that BCA improved apoptosis rates under iron overload, suggesting it helps on multiple cell-death fronts (since ferroptosis and apoptosis can intersect). BCA’s mechanism may involve upregulating antioxidant defenses or iron storage proteins, though the exact molecular target wasn’t fully elucidated in that abstract. The dual functionality of BCA (anti-catabolic and anti-ferroptotic) makes it particularly interesting. One could argue that any good antioxidant might also mitigate ferroptosis to some extent; however, not all antioxidants reduce iron levels, which BCA appears to do. The translational issue is whether systemic BCA could modulate iron in human joints maybe not directly, but reducing local inflammation might secondarily reduce iron accumulation (e.g., by reducing bleeding or cell turnover that releases iron).

Paeoniflorin (PAE) is a monoterpene glucoside from *Paeonia lactiflora* Pall. (white peony root) and *Paeonia suffruticosa* (moutan). It has notable anti-inflammatory and cytoprotective effects. Wu et al. (2025) explored paeoniflorin in an iron overload-induced OA cell model and *in vivo*. PAE significantly improved chondrocyte viability under high iron conditions, restored matrix gene expression, and reduced markers of oxidative stress. Mechanistically, PAE downregulated p53 (a pro-ferroptotic signal when activated) and upregulated SLC7A11 and GPX4. SLC7A11 is part of system Xc, which imports cystine for glutathione synthesis, and GPX4 is an enzyme that directly quenches lipid peroxides; together they are key inhibitors of ferroptosis. The fact that PAE boosted these suggests it helps chondrocytes resist ferroptotic death. Moreover, using Nutlin-3 (which activates p53) partially reversed PAE’s protective effects, indicating that the p53 pathway is indeed involved in PAE’s mechanism. In an *in vivo* model (likely similar to He et al.’s model or perhaps a systemic iron-dextran model), PAE-treated mice showed less subchondral bone loss and cartilage destruction, and had reduced iron deposits in joint tissues. Paeoniflorin is an interesting compound because it’s relatively abundant in peony (which is used in many TCM formulas), and it has known effects on the immune system (e.g., used in some Chinese arthritis medicines like TWP, total glucosides of peony). If it can be shown to specifically counter ferroptosis in joints, that adds a new dimension to its therapeutic profile. Its molecular weight and polarity (it’s a glucoside) give it decent oral availability, though how much reaches joints is unknown.

Salidroside (SAL), a phenylpropanoid glycoside from *Rhodiola rosea* L. (Hong Jing Tian), has antioxidant properties and potential to modulate cell death. Zhang et al. (2025) looked at salidroside in an LPS-induced chondrocyte inflammation model. While LPS is not a typical OA trigger, it induces acute inflammatory damage and can cause ferroptosis-like features in chondrocytes. Salidroside treatment significantly attenuated LPS-induced chondrocyte death and prevented the accumulation of intracellular iron and lipid ROS. It preserved the integrity of the cartilage ECM in explant cultures and promoted activation of the SIRT1/FOXO1 pathway. SIRT1/FOXO1 is associated with stress resistance and longevity; FOXO1 can induce antioxidant genes. By modulating this axis, SAL likely enhances the cells’ ability to cope with oxidative stress and thus ferroptosis. This study is limited to cellular analysis but gives mechanistic clues. Rhodiola extracts (which contain salidroside) are used as adaptogens, which aligns with the idea of boosting intrinsic defense pathways like SIRT1/FOXO1. Although not explicitly tested in an OA model, the findings justify further testing of salidroside *in vivo*, especially since it’s generally safe and orally bioavailable (being a small glycoside).

### 3.8 Regulation of OA-Related signaling pathways

OA pathophysiology involves numerous signaling pathways that regulate inflammation, cartilage metabolism, and subchondral bone remodeling. Among these, the PI3K/Akt/mTOR, NF-κB, and Wnt/β-catenin pathways are particularly prominent in mediating the effects of various stimuli on joint cells.

#### 3.8.1 PI3K/Akt/mTOR signaling pathway

**FIGURE 1:**
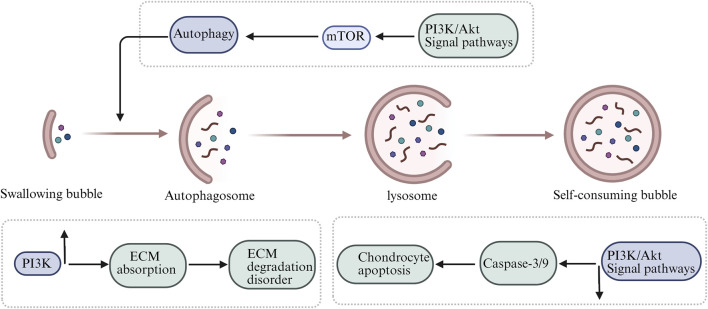
Schematic illustration of the PI3K/Akt/mTOR-related autophagy pathway in osteoarthritis, showing the relationships among autophagy, extracellular matrix degradation, and chondrocyte apoptosis.

The PI3K/Akt/mTOR pathway is a complex signaling network with multiple regulators and effectors and is essential for cartilage homeostasis (Sun et al., 2020). Previous analyses have suggested that the PI3K/Akt/mTOR pathway is downregulated in cartilage tissue from OA patients compared with normal cartilage (Rosa et al., 2011). Therefore, inhibiting the PI3K/Akt/mTOR pathway to restore intra-cartilaginous balance may alleviate OA-associated joint injury. Autophagy is a critical regulator of energy utilization and nutrient metabolism and serves as a cellular homeostatic mechanism that clears dysfunctional macromolecules and organelles (Chen et al., 2013). Autophagy failure leads to increased ROS production and mitochondrial dysfunction, and the autophagy-to-apoptosis switch has been implicated in chondrocyte progression toward OA (Duarte, 2015). The mTOR pathway is a key suppressor of autophagy and is centrally regulated by upstream PI3K/Akt signaling. Upregulation of mTOR in OA cartilage is associated with increased chondrocyte apoptosis and reduced expression of autophagy-related genes (Dalle Pezze et al., 2016). Studies have shown that cartilage-specific mTOR deletion enhances autophagy, reduces apoptosis, and alters intra-cartilage homeostasis in mice (Zhang et al., 2015). The potential mechanisms by which PI3K/Akt/mTOR signaling regulates OA progression are summarized in Figure 1.

Many TCM metabolites converge on this pathway. As mentioned earlier, quercetin, icariin, scutellarin, and others all showed impacts on PI3K/Akt/mTOR signaling in chondrocytes. Let’s highlight a few:Oroxin B (OB), a flavonoid glucoside from *Oroxylum indicum* (L.) Kurz (Bignoniaceae), has known anti-inflammatory and analgesic effects. Lu et al. (2022) demonstrated that OB (50 µM *in vitro*, and intra-articular injections in DMM mice) could reverse IL-1β induced changes in chondrocytes. It restored the levels of aggrecan and type II collagen while reducing MMP-3, MMP-13, and ADAMTS-5 production. Mechanistically, OB inhibited the IL-1β stimulated activation of PI3K/Akt/mTOR signaling in chondrocytes. In DMM model mice, OB injections led to less cartilage degradation and more normal expression of matrix components and MMP-13, aligning with the *in vitro* results. This suggests OB induces a pro-autophagic, anti-catabolic state by turning down mTOR. Oroxin B’s bioavailability is not well-known; being a glycoside, it might be metabolized into its aglycone or other forms in the body. The direct intra-articular approach in mice provided proof of concept. For translation, one might consider OB analogs or prodrugs that survive oral administration or again, local delivery for patients with early OA in a single joint.

Icariin (ICA) is a prenylated flavonol glycoside from *Epimedium brevicornu* Maxim. (Yin Yang Huo) noted for pro-chondrogenic and anti-inflammatory properties. Chen Y. et al. (202b) found that icariin (20 µM) pretreatment of SW1353 chondrocyte-like cells inhibited IL-1β–induced increases in MMP-3 and prevented the loss of type II collagen. It also restored autophagy-related proteins (ULK1, Beclin-1, LC3-II) that were suppressed by IL-1β. Importantly, icariin reduced the phosphorylation of PI3K, Akt, and mTOR, indicating an inhibition of the pathway, while upregulating ULK1, a key initiator of autophagy. In a DMM rat model, oral or ip icariin treatment led to reduced cartilage damage compared to controls, suggesting that it can reach the joint and exert effects *in vivo*(though the referenced text is about cells, the study likely had an *in vivo* component given the context). This supports the idea that icariin’s chondroprotective effect is linked to promoting autophagy and inhibiting excessive PI3K/Akt/mTOR signaling. One advantage with icariin is that it is relatively well-studied for bioavailability and metabolism; it does have limited oral absorption, but some *in vivo* studies used higher dosing to compensate. Also, derivatives like icaritin (the aglycone) might be more bioactive. At least one clinical trial in osteoporosis used an Epimedium extract, hinting at safety, but for OA specifically, more work is needed.

Scutellarin, another flavonoid (a glycoside of scutellarein) from *S. baicalensis* (or *Erigeron breviscapus* in some sources), was studied by Ju et al. (2021). In IL-1β–stimulated SW1353 cells, scutellarin increased type II collagen and SOX9 while inhibiting MMP-13. It also affected cholesterol metabolism genes (CH25H, CYP7B1) and inflammation (reducing IL-6), indicating broad effects on chondrocyte metabolism. Pathway analysis confirmed that scutellarin significantly suppressed PI3K/Akt/mTOR phosphorylation, aligning with the theme that inhibiting this pathway yields anabolic and anti-inflammatory benefits. Because SW1353 is a chondrosarcoma-derived cell line, one might critique that primary chondrocytes would be a better model; however, results likely translate qualitatively. Scutellarin is interesting because it’s present when baicalin is hydrolyzed (baicalin and scutellarin differ slightly in structure), and both are in *Scutellaria* roots. So one could surmise that *Scutellaria* extracts might deliver a combination of baicalin, scutellarin, etc*.*, hitting multiple targets. No *in vivo* data were in that particular study; future research should test scutellarin or its parent herb in animal models.

#### 3.8.2 NF-κB signaling pathway

**FIGURE 2:**
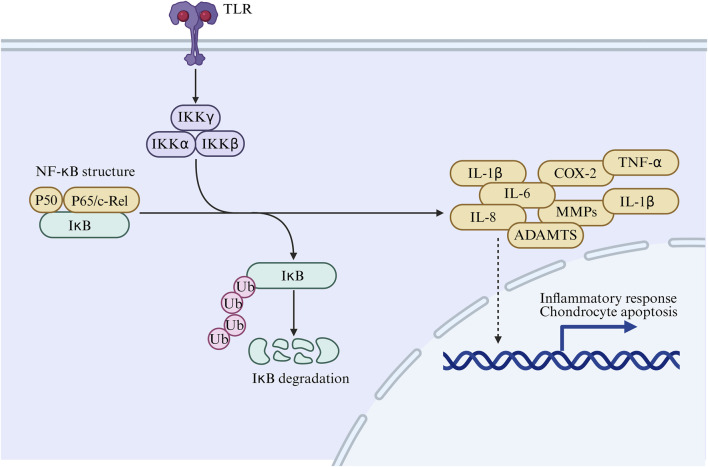
Schematic illustration of the TLR/NF-kB signaling pathway in osteoarthritis, showing IkB degradation, inflammatory mediator production, and chondrocyte apoptosis.

NF-κB is aberrantly activated in OA and functions as a pathogenic factor involved in multiple OA-related processes, including chondrocyte catabolism, cell survival, and synovial inflammation (Lepetsos et al., 2019). NF-κB directly or indirectly induces expression of matrix-degrading enzymes and other OA-related factors, coordinating abnormal cartilage catabolic programs (Kobayashi et al., 2013). NF-κB drives catabolic gene expression through response elements in the promoters of MMP-1, MMP-9, and ADAMTS-5, and promotes expression of key pro-inflammatory and OA-destructive mediators, including IL-1β, TNF-α, IL-6, COX-2, and PGE2 (Latourte et al., 2017). Potential mechanisms of NF-κB signaling in OA progression are summarized in Figure 2.

As we have seen, almost all compounds discussed have an NF-κB inhibitory effect one way or another. Polygonatum sibiricum Polysaccharides (PSP), although not a small molecule metabolite, polysaccharides from *Polygonatum* (Huang Jing) are active components often considered in TCM. Kuang et al. (2024) showed that these polysaccharides protected against knee OA in a DMM mouse model. They inhibited IL-1β–induced inflammatory responses in cultured chondrocytes and suppressed activation of the TLR2/NF-κB pathway *in vivo*. Mice treated with PSP had reduced cartilage degradation and lower serum levels of inflammatory cytokines. Polysaccharides generally have immunomodulatory effects, possibly by binding to receptors like TLRs or modulating gut microbiota. This study suggests that *Polygonatum* polysaccharides might act by blocking TLR2-mediated NF-κB activation, which is plausible given TLR2 can recognize damage-associated molecules in joint injury. As a high-molecular-weight component, PSP would likely not be absorbed intact but could exert effects in the gut or synovium if injected. The precise mechanism is less clear than for pure compounds, but NF-κB inhibition is central.

Wedelolactone, a coumarin from *Eclipta prostrata* (and *Wedelia chinensis*), was studied by Sun et al. (2024) Wedelolactone at micromolar levels inhibited IL-1β–induced IκB-α degradation, thereby preventing NF-κB p65 from entering the nucleus in chondrocytes. This led to marked reductions in inflammatory mediators (e.g., NO, PGE_2_) and MMPs. In a DMM mouse model, wedelolactone-treated mice had significantly less cartilage damage than controls. Wedelolactone’s mechanism might involve direct interaction with IKK (the kinase that phosphorylates IκB) or upstream signaling, as other studies suggest it can bind to kinases or transcription factors. The fact that it works *in vivo* in OA models is promising, especially since *E. prostrata* is a known anti-inflammatory herb. The limitation might be its pharmacokinetics: being a small lactone, it could be quickly metabolized, but perhaps frequent dosing or formulation can mitigate that.

Resveratrol (RSV), we revisit it one more time as Yi et al. (2020). Study in 2020 specifically looked at NF-κB in human chondrocytes. RSV at 50 µM inhibited IκB-α degradation and blocked NF-κB activation, which in turn suppressed downstream inflammation and MMP production. This aligns with numerous other findings that resveratrol is an NF-κB pathway inhibitor (it’s known to activate Sirtuin1, which can deacetylate p65, reducing its activity). The fact it was tested on OA patient-derived chondrocytes adds clinical relevance meaning even in the diseased state, resveratrol retained efficacy in cells. For translation, again, resveratrol’s challenge is delivery; perhaps local injection into joints via a hydrogel might be an approach, as systemic dosing might not concentrate enough in joints.

#### 3.8.3 Wnt/β-catenin signaling pathway

**FIGURE 3:**
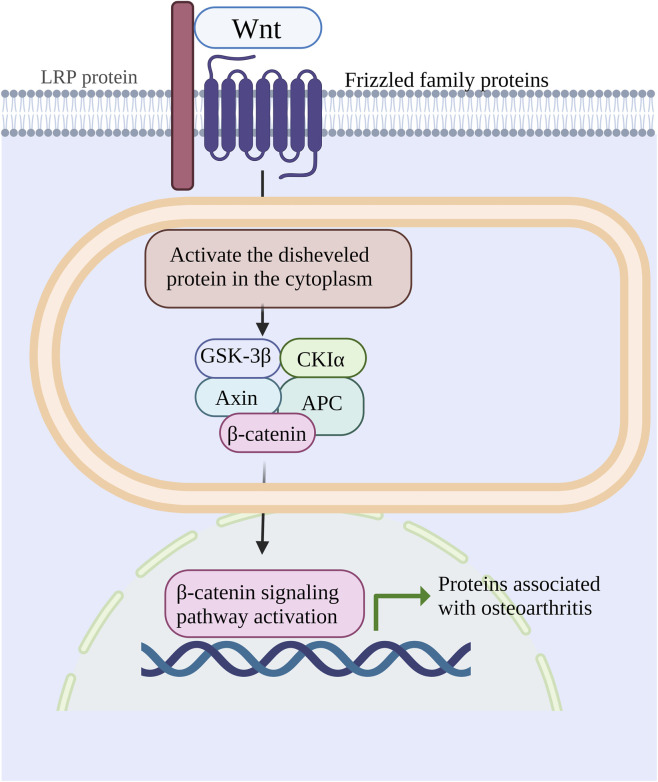
Schematic illustration of the Wnt/b-catenin signaling pathway in osteoarthritis, showing Wnt binding to Frizzled/LRP receptors, downstream b-catenin activation, and OA-related protein expression.

The Wnt/β-catenin pathway is a distinct signaling route involved in OA development and progression. In human genetics, gene alterations related to Wnt/β-catenin signaling have been regarded as susceptibility factors for OA (Zhou et al., 2017). The canonical Wnt pathway triggers intracellular signaling by regulating β-catenin levels and subcellular localization. In the absence of Wnt ligands, β-catenin remains stable and is degraded in a phosphorylation-dependent “destruction complex” involving Axin1/2, adenomatous polyposis coli (APC), Dishevelled proteins, and casein kinase I (CKI) (Yao et al., 2023). This complex phosphorylates specific residues at the C-terminal region of β-catenin (Wiese et al., 2018). When Wnt ligands bind to Frizzled receptors and co-receptors LRP5/6, the intracellular domains of these receptors interact with Dishevelled, APC, and Axin proteins, releasing β-catenin from the destruction complex and allowing its nuclear translocation (DeBruine et al., 2017). In the nucleus, β-catenin binds T-cell transcription factors and activates Wnt target gene expression. Sclerostin acts as a Wnt antagonist by competitively binding LRP5/6 and inhibiting Wnt signal transduction (Liu et al., 2022). Secreted frizzled-related protein-3 is a soluble inhibitor of Wnt/β-catenin signaling, and its variants have been associated with risk of hip OA; reduced inhibitor binding to Wnts increases Wnt/β-catenin activation (De Palma and Nalesso, 2021). Potential mechanisms of Wnt/β-catenin signaling in OA progression are summarized in Figure 3.

Traditional Chinese Medicine research into Wnt in OA has identified some compounds and polysaccharides that modulate this pathway. Achyranthes bidentata polysaccharides (ABPS), (Weng et al., 2014) found that ABPS promoted chondrocyte proliferation and matrix synthesis via activating Wnt/β-catenin. They isolated rat chondrocytes and treated with ABPS, observing upregulation of Wnt4, Frizzled-2, β-catenin, and Cyclin D1, along with increased nuclear translocation of β-catenin. This was associated with increased collagen II production. At first glance, activating Wnt might seem counterintuitive, but perhaps in that specific context, the chondrocytes were in a quiescent state and needed pro-proliferative signaling. Or it could be that short-term activation of Wnt is beneficial for anabolic repair. Notably, Achyranthes (Niu Xi) is traditionally used for strengthening bones and joints–maybe mild stimulation of Wnt could help cartilage regeneration. However, *in vivo*, chronic Wnt activation can be deleterious, so the timing and degree are key. The study did not mention an animal model, so evidence of benefit in actual OA via Wnt activation is lacking. It’s a reminder that pathways can have dual roles, and the context (healthy chondrocyte versus OA chondrocyte) may change the desired direction of modulation.

Icariin interestingly, in the context of Wnt (Zeng et al., 2014) showed that icariin inhibited IL-1β–induced increases in β-catenin in SW1353 cells and rat cartilage. It also reduced phosphorylated p38 and JNK levels. They noted that icariin’s suppression of MMP-13 was stronger than using specific inhibitors for p38, JNK, or β-catenin individually, hinting that its multi-pathway action (including Wnt/β-catenin inhibition) has synergistic effect. Another study by Zhu et al. (2022) went a step further: they used an icariin-loaded hydrogel with bone marrow stem cells in an OA model and found that it promoted chondrogenic differentiation via Wnt/β-catenin signaling. That sounds contradictory to the previous: one says icariin inhibits β-catenin to reduce catabolism, another says it uses Wnt activation to aid regeneration. Possibly, low-level or transient Wnt activation helps mesenchymal stem cells differentiate into chondrocytes (good), while chronic Wnt in mature chondrocytes causes matrix breakdown. Icariin might have the ability to modulate Wnt contextually. It’s also possible these studies looked at different readouts–one at MMP expression (where Wnt is bad), the other at differentiation markers (where Wnt is good up to a point). This underscores that Wnt signaling needs careful tuning in any therapy: complete blockade may impair repair, while overstimulation may worsen degeneration.

Artemisinin (ART), from *Artemisia annua* L, was shown by Zhong et al. (2018) to inhibit OA progression partly via antagonizing Wnt/β-catenin. Artemisinin reduced levels of IL-1β, IL-6, TNF-α, and MMP-13 in a rat OA model, consistent with general anti-inflammatory effects. It also enhanced chondrocyte viability and matrix production. At the pathway level, ART suppressed β-catenin accumulation in cartilage, suggesting it acts as a Wnt/β-catenin pathway inhibitor in this context. Artemisinin is best known as an antimalarial, but it also has anti-inflammatory and even anti-proliferative (in cancer) effects, often through affecting canonical Wnt signaling among others. Given its established use in humans (for malaria), repurposing it for OA could be of interest if efficacy is shown. Artemisinin derivatives or combination therapies might amplify effects (in malaria, artemisinin is often combined with other drugs; similarly, maybe in OA it could be combined with, say, a low-dose NSAID or a growth factor).

### 3.9 Limitations and future directions

Despite the extensive preclinical data summarized above, several important limitations and challenges must be acknowledged when translating these findings into clinical impact:

- Predominance of Preclinical Evidence: The majority of studies reviewed are confined to *in vitro* cell experiments or small-animal models. While these models are indispensable for mechanistic insights, they cannot fully replicate the complexity of human OA. For instance, isolated chondrocyte cultures or immortalized cell lines (like SW1353) may respond to compounds differently than chondrocytes in the native cartilage matrix or whole joint environment. Similarly, rodent models of OA (surgical or chemically induced) often represent post-traumatic or inflammatory extremes and may overestimate therapeutic effects. High-quality clinical trials validating these TCM metabolites in human OA are lacking. Future research should prioritize moving the most promising candidates (e.g., those with strong efficacy and safety in animals) into clinical testing. Small exploratory trials could measure biomarkers (like collagen breakdown products or inflammatory cytokines in synovial fluid) as proof-of-concept before larger efficacy trials.
- Dosing, Bioavailability, and Formulation Issues: Many active metabolites identified (flavonoids, polyphenols, saponins) suffer from poor oral bioavailability. This is due to factors like low absorption, rapid metabolism, or low stability *in vivo.* For example, quercetin and resveratrol have limited systemic availability, and icariin is extensively metabolized in the gut. Several studies achieved positive results by using routes like intra-articular injection or by using relatively high concentrations *in vitro* that might be difficult to attain in humans through oral dosing. This discrepancy raises concerns about the feasibility of replicating these results clinically. To address this, future work should include pharmacokinetic and pharmacodynamic studies for each candidate compound, and explore novel drug delivery systems. Promising strategies include nanoparticle encapsulation (to enhance joint delivery), conjugation with carriers (like hyaluronic acid or exosomes as seen with quercetin), and prodrug approaches. By improving local joint bioavailability, one can potentially use lower systemic doses, improving safety margins.
- Variability and Reproducibility: There is noticeable variability in experimental designs across studies different induction methods, different outcome measures, and sometimes conflicting results (e.g., icariin’s dual effect on Wnt signaling). This variability can make it hard to compare compounds or to identify which pathways are truly central. Moreover, some studies lacked comprehensive controls (for instance, not all studies included a positive control such as a known anti-OA drug, which would help benchmark the efficacy of the metabolite). To strengthen the evidence base, standardized models should be considered. For example, testing multiple top compounds under the same conditions (same animal model, same outcome metrics) could reveal relative effectiveness and potential synergistic combinations. Additionally, ensuring rigorous controls (vehicle controls, dose-response, and ideally a reference compound like an NSAID or DMOAD candidate) would make results more robust and clinically meaningful.
- Mechanistic Complexity Multi Target Effects: TCM metabolites often have pleiotropic actions, hitting multiple pathways (which is arguably their strength). However, this also complicates mechanistic dissection. It can be challenging to pinpoint the primary mechanism responsible for therapeutic effects. For instance, a flavonoid may concurrently reduce inflammation, oxidative stress, and modulate growth factor signaling. Traditional single-target assays may not capture this network effect. Embracing systems biology approaches, such as transcriptomic or proteomic profiling of treated vs. untreated joint tissues could help map the network of changes induced by a given compound, identifying key “nodes.” Network pharmacology analyses, which are increasingly used in TCM research, could predict synergistic targets and help focus future mechanistic studies on the most pertinent pathways rather than examining one pathway at a time. Also, the “double-edged sword” nature of certain pathways (like Wnt or autophagy) needs careful investigation to determine optimal modulation levels rather than blanket inhibition or activation.
- Safety and Off-Target Effects: An often under-reported aspect in preclinical studies is safety. While many of these natural compounds have been used in traditional contexts (suggesting general safety), potency at therapeutic doses could bring side effects. For example, systemic immune suppression from NF-κB inhibitors or endocrine effects from phytoestrogens like biochanin A. None of the reviewed studies reported severe adverse effects in animals, but animals were often young and healthy aside from induced OA. As these therapies move forward, evaluating their safety in aged animals or in combination with other medications will be important, since OA patients are often older and on polypharmacy. Furthermore, ensuring that long-term inhibition of certain pathways (like mTOR or NF-κB) does not predispose to infections or metabolic disturbances will be key. For polysaccharide-based treatments, immunogenicity should be assessed.

Looking ahead, future research priorities should include:

- Clinical Translation Steps: Identify leading compounds (or a combination) with strong preclinical evidence and conduct dose-finding and toxicity studies in larger animals (like rabbits or canines that naturally develop OA). Subsequently, design early-phase clinical trials focusing on safety, pharmacokinetics, and biomarker efficacy (e.g., changes in inflammatory cytokines, imaging of cartilage thickness via MRI).
- Advanced Drug Delivery: Invest in nano-formulations, hydrogels, or sustained-release intra-articular injections for delivering these metabolites directly to joint tissues. For instance, liposomal formulations of resveratrol or baicalin that could be injected monthly into an arthritic knee, or oral nanoparticles that preferentially home to inflamed joints. Targeted delivery could dramatically enhance local concentrations while minimizing systemic exposure.
- Combination Therapies: Considering the multifactorial nature of OA, it may be unrealistic that a single compound (natural or otherwise) will be sufficiently disease-modifying. Future studies should explore combining TCM metabolites with conventional treatments. Additionally, integrating these compounds with non-pharmacological interventions (e.g., exercise or cartilage scaffolds) could yield holistic approaches (for example, a scaffold impregnated with icariin to repair focal cartilage defects).
- Biomarkers and Patient Stratification: OA is heterogeneous; some patients have more inflammatory phenotypes (where NF-κB inhibitors might shine), others have more metabolic or aging-related OA (where antioxidants or ferroptosis inhibitors might be key). Future research should aim to correlate the mechanisms of these TCM compounds with specific OA patient subsets. Developing biomarkers (from synovial fluid or serum) that indicate oxidative stress level, inflammatory status, or cartilage turnover could help identify which patients might respond best to a given therapy. This stratified approach could improve the signal detection in trials and ultimately patient outcomes.

In conclusion of this section, while the journey from bench to bedside is non-trivial, the rich arsenal of TCM-derived metabolites provides a promising starting point for OA drug discovery. By addressing the above limitations with rigorous, well-designed studies and innovative translational strategies, we can better assess which of these multi-target agents holds real potential to become part of the future OA therapy toolbox.

### 3.10 Conclusion

OA is a multifactorial disease, and the multi-target pharmacology of TCM-derived metabolites offers a compelling paradigm to tackle its complexity. The evidence reviewed herein demonstrates that a diverse array of compounds isolated from TCM botanical drugs can beneficially modulate the pathological processes of OA. These metabolites ranging from flavonoids like quercetin and kaempferol, to polyphenols like resveratrol and pterostilbene, terpenoids like ginsenoside Rg1 and artemisinin, alkaloids like dendrobine, and polysaccharides from Achyranthes or Polygonatum, exert convergent protective effects on joint tissues. They suppress inflammatory cascades, restore redox balance, preserve cartilage matrix by downregulating catabolic enzymes, prevent chondrocyte death (whether by apoptosis, senescence, or ferroptosis), and even recalibrate the gut joint axis. Notably, these mechanisms are intertwined; for example, by inhibiting NF-κB, a compound can simultaneously reduce inflammation and the production of MMPs, indirectly protecting cartilage while improving symptoms.

However, translating these insights into therapies will require surmounting the challenges discussed. Most importantly, rigorous clinical investigation is needed to validate efficacy and safety in humans. Encouragingly, some TCM compounds (like curcumin, used in turmeric) have already entered clinical trials for OA, and novel ones are on the horizon. With continued research that bridges traditional knowledge and cutting-edge biomedical science, we anticipate that TCM metabolites will contribute to the development of new, more effective treatments for OA. These could take the form of new drugs derived from natural templates or as adjuvants that complement existing treatments to enhance overall outcomes. Ultimately, the integration of such multi-target strategies could mark a shift from merely alleviating OA symptoms to actively modifying the disease course, offering patients improved joint function and quality of life.

## Data Availability

The original contributions presented in the study are included in the article/Supplementary Material, further inquiries can be directed to the corresponding authors.
